# From Raw Signals to Understood States: A Perspective on Neuro-Symbolic and LLM-Driven Intelligent Sensing of Human Affective and Cognitive States

**DOI:** 10.3390/s26196066

**Published:** 2026-09-24

**Authors:** Christos Papakostas, Christos Troussas, Akrivi Krouska, Cleo Sgouropoulou

**Affiliations:** Department of Informatics and Computer Engineering, University of West Attica, 12243 Egaleo, Greece; cpapakostas@uniwa.gr (C.P.); akrouska@uniwa.gr (A.K.); csgouro@uniwa.gr (C.S.)

**Keywords:** intelligent sensing systems, multimodal sensor fusion, affective computing, cognitive-state recognition, neuro-symbolic AI, large language models, explainable AI, fuzzy learner modeling, wearable sensors, human–AI collaboration

## Abstract

Intelligent sensor technologies now provide unprecedented multimodal access to human affective and cognitive processes, spanning physiological, ocular, facial, kinematic, ambient, and behavioral streams. Yet, despite dramatic advances in acquisition and deep representation learning, the pipeline from raw signals to psychologically meaningful, actionable understanding remains fragile. Deep-only architectures excel at pattern extraction but struggle with contextual reasoning, uncertainty communication, and human-facing explanation; symbolic-only frameworks resist noisy, high-dimensional streams. This perspective argues that the field’s next inflection point lies not in richer sensors alone but in the interpretive layer that turns signals into states. We propose an integrative view in which neuro-symbolic fusion couples continuous sensor evidence to psychologically grounded symbolic primitives, LLMs act as auditable semantic reasoners rather than opaque classifiers, and explainability is treated as a design constraint rather than a post hoc addition. Synthesizing a decade of work on affective computing, fuzzy learner modeling, neuro-adaptive multimodal interaction, and explainable human–AI collaboration, we motivate a research agenda organized around grounded representations, uncertainty-calibrated LLM reasoning, and reflexive explanation. The aim is a class of sensing systems whose intelligence is measured not by accuracy alone but by the quality of the states they help humans understand.

## 1. Introduction

Two parallel trajectories have shaped the last decade of intelligent sensor research. The first is a story of acquisition: wearable biosensors, high-resolution cameras, low-cost thermal and mmWave arrays, eye-tracking modules, ambient microphones, and inertial units have made it routine to observe human physiology and behavior at temporal and spatial resolutions that were, until recently, confined to specialist laboratories [1,2,3,4]. The second is a story of representation: deep neural architectures, self-supervised pretraining, and multimodal transformers have provided the modeling machinery to map noisy, high-dimensional streams onto discrete labels or continuous affective/cognitive dimensions [5,6,7,8,9]. Taken together, these advances are typically described as making intelligent sensing systems “ready” for pervasive deployment in healthcare, education, workplace analytics, and immersive human–computer interaction.

Our position, rooted in our longitudinal work on affective computing and adaptive modeling [10,11,12,13,14] and seminal contributions from the wider literature [15,16,17,18,19,20,21,22,23], is that this readiness narrative is premature—not because the sensors are insufficient but because the interpretive layer between the signal and the decision has not matured at the same pace. Intelligent sensing systems that observe humans still routinely produce artifacts of the form “class k at time t, with confidence p”, where the class label is drawn from a coarse discrete ontology (e.g., “engaged/bored”, “happy/neutral/stressed”), where the confidence p is poorly calibrated, and where the pathway from the physiological or behavioral evidence to the classification decision is neither auditable by a domain expert nor accessible to the person being sensed. This is the signal-to-meaning gap: the distance between what our sensor arrays can measure and what a psychologist, clinician, teacher, or the user themselves would recognize as an understanding of that person’s state.

Recent scholarship has begun to name this problem from several angles. Systematic reviews of physiological cognitive-state recognition emphasize the persistent lack of contextual grounding and cross-domain transferability [3]. Interdisciplinary scoping work mapping EEG metrics onto affective and cognitive models highlights the missing bridge between neural signatures and psychological constructs [24]. Multimodal biosignal transformers achieve state-of-the-art scores yet remain difficult to interpret at the individual-decision level [9]. In educational and workplace settings, multimodal analyses of learner behavior recover rich state trajectories but stop short of psychologically meaningful explanations that a teacher could act on [25,26,27]. A recent perspective on multimodal-sensing-enabled LLMs for emotional regulation frames the same tension explicitly: as sensor pipelines feed LLM stacks, the challenge is no longer signal quality but the semantic and epistemic quality of the interpretation [28]. We frame this not as an established, empirically settled fact but as a central synthesizing hypothesis—a defensible perspective inferred from recurring structural limits documented across empirical sensor literature.

This perspective advances a personal thesis in three propositions, developed in the remainder of this paper. (1) Interpretive layer is becoming a more critical operational bottleneck along with existing sensor hardware issues. Motivated by the recurring cross-domain degradation in the literature, we hypothesize that the addition of modalities and encoder depth alone yields diminishing returns under real-world distribution shifts, and thus, a critical frontier of marginal gain now resides in the semantic reasoning layer (Section 2 and Section 3). (2) The main conceptual contribution of this perspective is an integrative architectural synthesis bridging the signal-to-meaning gap across three previously isolated paradigms: coupling continuous multimodal sensor streams to psychologically grounded fuzzy primitives, deploying LLMs strictly as auditable semantic reasoners over structured symbolic evidence rather than end-to-end black-box predictors, and establishing a closed-loop reflexive dialog protocol allowing real-time user contestation and symbolic state repair (Section 4, Section 5 and Section 6). (3) Explainability must be a design constraint, not an afterthought. For sensing systems that observe humans, explanation is not a compliance add-on: it is the mechanism through which the interpretation is stabilized, contested, and trusted (Section 6).

We then illustrate the argument with three vignettes drawn from our own and adjacent research programs—adaptive learning, clinical monitoring, and immersive human–computer interaction (Section 7)—and close with a research agenda that reframes what “intelligent” should mean for the next generation of sensor systems (Section 8). We do not attempt a systematic review; instead, in the spirit of the perspective format, we seek a grounded, defensible reframing of where the field’s leverage now resides. To ensure methodological transparency, a purposive conceptual sampling was followed to select the literature from the major computational and psychophysiological repositories. The inclusion criteria for the works were: (i) multimodal acquisition of physiological, ocular or behavioral signals targeting affective or cognitive states; (ii) explicit engagement with neuro-symbolic, fuzzy, or foundation-model reasoning paradigms; and (iii) technical or educational deployments emphasizing interpretability, calibration or operational failure modes under distribution shift. Foundational historical papers were retained only as a basis for long-standing theoretical constructs. Figure 1 summarizes, at a glance, the contrast between the conventional deep-only pipeline and the three-part interpretive architecture that this perspective advances.

## 2. What We Can Now Sense About Humans: Landscape and Limits

We begin by summarizing, without exhaustiveness, the modalities that a contemporary intelligent sensing system can plausibly draw upon for human affective and cognitive-state estimation. Our goal in this section is not to catalogue devices but to characterize the shape of the evidence they produce, so that the interpretive gap identified in Section 3 becomes concrete.

### 2.1. Physiological and Biosignal Streams

Wearable and ambulatory biosignal sensors—electrodermal activity (EDA), photoplethysmography (PPG), electro-cardiography (ECG), electroencephalography (EEG), respiration bands, and skin-temperature sensors—provide continuous access to autonomic and central nervous system correlates of arousal, valence, workload, and stress [1,3]. Systematic reviews of physiological cognitive-state recognition report that machine learning strategies over such signals now routinely exceed chance-level performance across attention, workload, engagement, and fatigue detection tasks in laboratory conditions [3]. Multimodal fusion of physiological channels—for example, via differential multimodal transformers over biosignals—pushes benchmark scores further, particularly when temporal and cross-modal dependencies are modeled jointly [9]. To clarify the complete processing chain from raw acquisition to downstream inference, explicit operationalization of the physical nature of these biosignals is required. Central neurodynamic monitoring at the sensory front-end is based primarily on electroencephalography (EEG), functional near-infrared spectroscopy (fNIRS) recording microvolt fluctuations (μV), and localized optical blood-oxygenation changes respectively. In differential biosignal architectures, raw multichannel time series are temporally band-pass filtered (usually 0.5–50 Hz) and spatially montaged with reference to eliminate ocular, cardiac, and muscular artifacts (e.g., common average or surface Laplacian). Then, temporal dynamics are decomposed into discrete spectral power bands, namely frontal theta (4–8 Hz), parietal alpha (8–12 Hz) and beta (13–30 Hz), using short time Fourier transforms (STFT) or continuous wavelet transforms. Topological electrode arrays allow for differential analysis like frontal alpha asymmetry (motivational valence) and phase-locking value (PLV) connectivity across frontoparietal networks in the spatial domain. These biosignal features are directly linked to different executive functions like working memory load (frontal theta synchronization), sustained vigilance, attentional allocation, and mental fatigue (alpha desynchronization and power migration). The raw voltage variations are fed into differential transformers, which are associated with the fuzzy cognitive primitives presented in our framework.

Beyond feature extraction, EEG has attracted increasing attention as an interpretive bridge between neural dynamics and psychological constructs, with scoping reviews mapping specific spectral and connectivity metrics onto established affective and cognitive models [24]. Even the cognitive impact of interacting with an LLM has been characterized using EEG-based analyses of problem-solving and decision-making, illustrating how sensor-side and AI-side pipelines are becoming co-dependent objects of study [29].

### 2.2. Visual, Ocular, and Facial Streams

Camera-based sensing recovers facial expressions, head and gaze dynamics, micro-motions, and—in stereoscopic or depth configurations—postural and gestural cues. Classical work in multimodal affect detection established that fusing conversational cues, gross body language, and facial features substantially improves affect estimation over unimodal baselines, an insight that continues to organize contemporary systems [7]. More recent architectures combine RGB video with mmWave radar to recover micro-motion patterns that are invisible to conventional cameras but that carry rich affective and psychological information [4]. Multimodal biomarker frameworks such as MEmoR argue that emotion recognition for people analytics is best framed as an aggregation over affective biomarkers spanning face, voice, and physiology [30]. In learning environments, vision–language models are being evaluated for their ability to infer engagement directly from classroom or webcam footage, replacing bespoke engagement classifiers with foundation-model reasoning over visual streams [31].

### 2.3. Behavioral, Kinematic, and Ambient Streams

Beyond physiology and vision, kinematic and ambient modalities have expanded the observable surface of human state. On-body inertial sensors have been shown to enable affect identification from gait alone, using smart devices commonly present in the wild [32]. Ambient acoustic and environmental sensors, combined with wearables, extend affective and cognitive estimation from controlled laboratory settings into everyday life [1]. Wearable multimodal architectures—of which our group’s NAMI framework is representative—integrate biosignals with contextual streams to support neuro-adaptive human–computer interaction in situ [21]. Personalized multimodal signal processing has similarly been developed for augmented-reality environments, where inertial, ocular, and interaction streams must be fused in real time to modulate the user experience [22].

### 2.4. Textual and Conversational Streams

A fourth, less classically “sensor-shaped” stream has become central to human-state estimation: text and conversation. In learning and clinical settings, natural-language interactions constitute an interpretable—and increasingly always-on—behavioral signal. Deep learning-based sentiment analysis over social media text established that pretrained word embeddings materially improve affect estimation from user-generated content [33]. More recent fuzzy-weighted sentiment recognition tailored to educational text-based interactions demonstrates that domain-adapted textual analytics can complement physiological and behavioral signals, particularly when the text itself is embedded in an instructional dialog [12]. Multimodal E-learning systems now routinely combine textual and visual streams via deep fusion for joint emotion and cognition detection [5].

### 2.5. The Limits of a Signal-First View

Viewed side by side, these modalities describe a striking range of what modern sensor systems can measure about a person: neural dynamics, autonomic tone, gaze and expression, gait, micro-motion, language use, and interactional context. Recent systematic reviews of wearable multimodal affective computing [1] and of state-of-the-art multimodal emotion recognition [8] confirm that the modality space has largely stabilized; new work now aggregates and recombines rather than fundamentally expands. Comprehensible-AI treatments of multimodal state detection make the same observation explicit: the field’s frontier is no longer what to sense but how to interpret what has already been sensed [34].

Two limits of a signal-first view then become visible. First, the outputs of even the strongest current pipelines remain in the shape of classifications or scalar regressions over pre-specified affective/cognitive labels; a psychologist would recognize few of them as an understanding of the person’s state and fewer still as an explanation that person could contest or refine. Second, evaluation is dominated by benchmark-level accuracy on constrained datasets, which offers no guarantee that the recovered state is contextually valid, temporally stable, or interpretable to a downstream human user [35,36]. It is these two limits that the remainder of this paper takes as its starting point.

To synthesize the multistream landscape reviewed above and clarify the evidential chain from sensor acquisition to interpretive targets, Table 1 categorizes the primary human sensing modalities, their underlying raw signals, temporal/spatial resolutions, target constructs, and operational bottlenecks.

## 3. The Interpretive Dimension: Why Sensor Scaling Requires Downstream Reasoning

We state our main thesis as a synthesis hypothesis: the primary rate-limiting bottleneck for deployable human-state understanding has become interpretive and semantic, in addition to the continuing essential advances in physical sensing and signal conditioning. We do not claim that sensor-level challenges are solved but rather stress that improvements in raw sensing cannot by themselves bridge the semantic gap.

### 3.1. Contextual Brittleness of Deep-Only Pipelines

Deep, end-to-end pipelines trained to map raw multimodal sensor windows onto affective or cognitive labels are, in our reading of the literature, powerful but contextually brittle. They excel when the deployment distribution matches the training distribution and degrade sharply when task, user, sensor placement, or ambient conditions shift [3,8]. This brittleness is not, as sometimes framed, a matter of “more data will fix it”; it is a structural consequence of representations that are optimized for label prediction rather than for capturing the semantic and contextual scaffolding that gives a physiological or behavioral pattern its psychological meaning. Independent evidence for this diagnosis comes from studies of interpretation drift under label noise, which show that even nominally state-of-the-art explainable models produce unstable explanations as annotations become imperfect—a chronic feature of affective and cognitive datasets [37]. Related work on the consistency of explanations across model instances shows that architecturally similar deep models can support incompatible interpretations of the same input [38].

Comprehensive computational-modeling perspectives on human cognition make the same point in stronger form: cognition, as psychologists model it, is structured by symbolic, hierarchical, and dynamical constraints that pure end-to-end deep networks do not natively encode [39]. When we ask a deep multimodal encoder to output a “cognitive state”, we are, in effect, asking it to compress those structural constraints into a scalar or a class index—and then act as if that scalar carried the full weight of the psychological construct. While scaling encoder architectures and increasing sensor arrays bring clear benefits in controlled benchmarks, empirical reviews increasingly show performance plateaus and severe degradations when moving across subjects, tasks, and unconstrained environments [3,8]. We interpret these repeated empirical findings in our conceptual synthesis as a sign of diminishing returns from sensory scaling alone, uncoupled from downstream contextual reasoning, rather than as evidence of final sufficiency of sensory technology.

### 3.2. Symbolic Frameworks That Do Not Scale to Raw Streams

The mirror-image failure mode is equally instructive. Purely symbolic frameworks—expert systems, rule-based cognitive diagnosers, formal cognitive architectures—encode precisely the kind of psychologically grounded structure that deep pipelines lack and produce interpretations that are auditable, contestable, and pedagogically or clinically actionable [40,41]. Our own line of work on cognitive-diagnostic modules based on repair theory [13], on the representation of generalized cognitive abilities in adaptive learning environments [14], and on the automated symbolic reasoning of learner cognitive states via classification analysis [15] illustrates the kind of interpretive traction that symbolic frameworks bring—traction that pure deep pipelines routinely lack.

The reciprocal weakness is well documented: symbolic systems do not scale to noisy, high-dimensional, temporally rich sensor streams without either heavy manual encoding or a learned front-end. A recent comprehensive review of neuro-symbolic AI for robustness, uncertainty quantification, and intervenability names this trade-off explicitly and argues that neither pole is, on its own, sufficient for real-world reasoning under uncertainty [42].

### 3.3. The State Is Semantic, Temporal, and Context-Bound

A useful way to summarize the two failure modes above is that they mismatch the shape of a human affective or cognitive state. Such a state is not a scalar and not a static label. It is a semantic construct—“confused about a specific concept”, “anxious about an upcoming assessment”, “engaged but fatigued”—that lives in a context (a task, a relationship, an environment), that unfolds over time, and that is inseparable from the reasons a downstream human user would offer for adopting it as a description. Multimodal analyses that combine emotional and cognitive states during problem-solving illustrate this vividly: the interpretive payoff arises not from any single modality but from the joint semantic reading that combines evidence across streams [26,27]. Recent work on self-supervised multimodal learning for cognitive-state inference from wearable streams similarly frames the challenge as one of learning representations that support downstream inference rather than isolated classification [2].

Contemporary comprehensive-AI treatments of multimodal state detection make the same point with different vocabulary: the requirement is not simply detection but comprehensible detection, in which the state assignment is legible to, and contestable by, a human interpreter [34]. In the language of user modeling, the target is not a label but a learner (or user) model—a structured, updatable, symbolic representation of the person that a system can reason with and a human can inspect [13,14]. Even in adjacent domains such as intelligent industrial monitoring, the same lesson has been drawn: sensor-driven pipelines that produce only opaque scores struggle in deployment relative to those that produce structured, semantically grounded outputs [43,44].

The consequence, for our argument, is clear. The pipeline from raw signals to understood states must contain a layer that is not simply another neural block. It must contain a layer that is semantic, symbolic, and context-aware. Section 4 and Section 5 describe two complementary components of such a layer: neuro-symbolic fusion and LLMs as semantic reasoners.

## 4. A Neuro-Symbolic Foundation for Human-State Sensing

Neuro-symbolic AI (NS-AI) has re-emerged over the last several years as a principled response to the neural/symbolic dichotomy identified in Section 3. Recent comprehensive reviews describe NS-AI as the fusion of learned neural representations with structured symbolic reasoning, with the joint objective of preserving the flexibility of the former and the interpretability, compositionality, and controllability of the latter [40,41,42]. Our claim in this section is that NS-AI provides the natural architectural foundation for human-state sensing—and, further, that many of its promised benefits are amplified, not attenuated, when the sensor stream is human and the state is affective or cognitive.

### 4.1. Grounding Sensor Evidence in Symbolic Priors

The core NS-AI move—grounding continuous evidence in symbolic priors—has been operationalized across sensing domains beyond human-state estimation. Neuro-symbolic fusion of Wi-Fi channel measurements with passive-radar reasoning demonstrates how symbolic priors can transfer inter-modal knowledge and stabilize interpretation under sparse or noisy conditions [45]. Adaptive multistage sensor fusion under a neuro-symbolic framework has been shown to improve multimodal ranging in adverse weather, precisely by constraining the neural encoder with symbolic weather models [46]. Scalable neural-symbolic stream fusion architectures have been proposed for large-scale streaming settings, showing that the coupling can be made to scale [47]. In cyber-deception domains, neuro-symbolic fusion is being explored for cognitive threat reasoning, in which raw telemetry is coupled to structured threat ontologies [48,49].

Our own recent work extends this line into semantic parsing itself: a hybrid neuro-symbolic pipeline for coreference resolution coupled with AMR-based semantic parsing shows how a neural front-end can be constrained by a symbolic semantic representation to produce interpretations that are both accurate and legible [19]. That result is directly relevant to human-state sensing, since the interpretive target—a state attributed to a person—is itself a semantic object over which symbolic operations (aggregation, revision, contradiction detection, explanation) must be defined.

### 4.2. Fuzzy Affective and Cognitive Primitives

A specific class of symbolic primitives is particularly well-suited to human-state sensing: fuzzy representations of affect and cognition. Affective and cognitive constructs rarely admit crisp membership functions; a learner is not “confused” or “not confused” but rather partially confused, over particular concepts, with a graded and time-varying intensity. Fuzzy set theory and fuzzy cognitive maps have historically provided a compact language for such graded, context-conditioned states. Independent recent proposals for neuro-symbolic affective-aware personalization in virtual learning environments demonstrate the general viability of this coupling at scale [50], and the CEREBRAL framework operationalizes neuro-symbolic multimodal emotion recognition with psychological constraints and metacognitive reasoning as an important existence proof [51]. Comprehensive treatments of neuro-symbolic AI foreground exactly the intervenability and uncertainty-quantification properties that fuzzy primitives make actionable [40,41,42], and interdisciplinary scoping of EEG-to-psychological-construct mappings supplies the sensor-side grounding [24]. Our own applications range across educational settings—from fuzzy cognitive-map-based policy modeling [16] to fuzzy memory networks that stabilize contextual reasoning in LLM-based educational dialog [17] and to fuzzy-weighted sentiment recognition for educational text interactions [12].

The coupling we advocate is straightforward in principle and non-trivial in practice: continuous sensor evidence (EEG spectral features, EDA phasic responses, gaze fixations, gait descriptors, textual sentiment) is mapped, via a learned neural front-end, onto graded membership values over psychologically motivated fuzzy variables (arousal, valence, engagement, cognitive load, confusion-about-concept-C). These fuzzy variables then serve as the substrate over which symbolic reasoning—cognitive diagnosis over learner models drawn from repair-theory traditions [13], structured cognitive-ability aggregation [14], and classification-based cognitive-state inference [15]—is performed. To this end, the mapping from the extracted biosignal features to the fuzzy primitives is based on parameterized trapezoidal and Gaussian membership functions, $\mu_{A}\left(x\right)\in \left[0,1\right]$, which are directly anchored in established psychological models. In particular, we derive cognitive load from Sweller’s Cognitive Load Theory via frontal theta synchronization (4–8 Hz) and pupillary dilation, affective arousal and valence from Russell’s Circumplex Model via phasic EDA peaks and frontal alpha asymmetry, and operationalize confusion from D’Mello and Graesser’s cognitive-disequilibrium model when high persistence is accompanied by physiological arousal and keystroke latencies. From a methodological point of view, the parameters of membership functions (thresholds, centers and spreads) are determined and validated according to a three-level calibration protocol allowing a trade-off between theoretical validity and inter-individual variability. First, the baseline bounds and initial geometric centroids are initialized using normative values reported in the psycho-physiological literature (e.g., canonical band power thresholds and tonic EDA recovery intervals) [3,24]. Second, to accommodate idiosyncratic physiological baselines, inputs are normalized to individual dynamic ranges through an initial resting-state acquisition phase to shift membership centroids to relative individual dynamic ranges. Third, supervised data-driven optimization is employed to fine-tune and empirically validate parameters, constrained against validated ground-truth affective benchmarks and expert annotations, thus ensuring robust boundary alignment while avoiding arbitrary parameter fitting.

Importantly, this fuzzy layer acts as an active error-containment barrier before representations reach the reasoning stage, as front-end neural extractors are still susceptible to sensor variability and domain shift. The first stage is temporal integration and hysteresis bands, which smooth raw sensor spikes by exponential moving averages. This avoids transient motion artifacts or short electrode disconnections from producing spurious changes in membership degrees. Second, dynamic SQIs such as spectral signal-to-noise ratios in EEG or electrode contact impedance modulate membership weights, automatically down-weighting degraded streams. Third, ontological boundary constraints provide domain-level physiological consistency, rejecting contradictory or physiologically implausible states (e.g., baseline autonomic quiescence paired with extreme panic) as acquisition faults rather than valid cognitive interpretations.

Comprehensible-AI treatments of multimodal state detection converge on the same architectural pattern from a different starting point [34], and computational-modeling perspectives on human cognition supply the psychological constraints against which the fuzzy layer must be grounded [39]. Affective-computing surveys in intelligent tutoring reinforce that this structured, grounded route is what the pedagogy actually needs [10,25].

### 4.3. From Stream Fusion to Symbolic Fusion

An important consequence of adopting a neuro-symbolic foundation is that the locus of fusion shifts. In signal-first pipelines, fusion is typically an operation over feature maps or embeddings—early, mid, or late fusion of encoder outputs [8,9]. In a neuro-symbolic architecture, the more decisive fusion happens at the symbolic layer, where evidence from disparate modalities is combined against a shared, psychologically meaningful ontology. This has three practical consequences.

First, the addition of a new modality no longer requires retraining a joint encoder; it requires a mapping from the new stream onto the existing symbolic primitives. Second, disagreement between modalities becomes semantically inspectable: a mismatch between facial-affect evidence and physiological-arousal evidence becomes a symbolic contradiction that can be surfaced, rather than a numerical inconsistency absorbed silently into the loss. Third, evidence-to-state calibration can be performed at the symbolic layer, aligning the system’s expressed confidence in a state with the psychological and clinical stakes of asserting it—a property that is essential in the settings we consider in Section 7 and that is difficult to obtain from purely neural pipelines [35,42].

Neuro-symbolic information fusion for explainable retrieval-augmented generation, developed in the context of mental-health applications, illustrates a further extension of this shift: the symbolic layer becomes the substrate over which explanation itself is generated and audited [52]. We return to this point in Section 6.

Table 2 compares representative neuro-symbolic, fuzzy, and LLM-driven affective architectures along primary design dimensions to clarify the conceptual and architectural positioning of our framework in relation to recent foundational milestones.

To maintain clear epistemic boundaries and sharpen the original conceptual positioning of this perspective, we explicitly distinguish our proposed framework from existing neuro-symbolic, affective-computing, foundation-model, and XAI frameworks (Table 2). Contemporary neuro-symbolic architectures (e.g., CEREBRAL [51], Olaniyan & Wario [50]) embed psychological constraints or expert rules but are mainly static, feed-forward constraint-satisfaction engines without dynamic, human-interpretable linguistic justification and interactive state correction. In contrast, recent multimodal-LLM affective pipelines (e.g., Yu et al. [28], Liao et al. [53]) utilize generative capabilities for affective dialog or post hoc rationale generation, but they either skip explicit intermediate symbolic abstractions, or they depend on uncalibrated token heuristics that are still susceptible to semantic drift and confabulation when faced with noisy biosignal inputs. The original conceptual contribution of this work is not in proposing isolated neuro-symbolic primitives or evaluating generic foundation models but rather in their unified three-tier synthesis: (i) an intermediate fuzzy grounding layer, as an active error-containment barrier between noisy continuous sensors and semantic representations; (ii) an uncertainty-calibrated, bounded LLM reasoner, operating strictly over auditable symbolic evidence tuples; and (iii) a closed-loop reflexive dialog protocol, whereby user contestation directly fuels symbolic state repair in the underlying user model, rather than producing ephemeral conversational adjustments. The main research agenda developed here is to validate this end-to-end integration over real-time, high dimension sensing deployments.

## 5. Large Language Models as Semantic Reasoners over Sensor Streams

The second component of the interpretive layer we advocate is a class of models that has, until recently, been treated as tangential to sensor systems: large language models. Our claim is not that LLMs should replace domain-specific sensor front-ends—they should not—but that they should be repositioned in the pipeline. Specifically, we argue that the interpretive value of LLMs for intelligent sensing systems lies not in their use as classifiers over embeddings but in their use as semantic reasoners over structured symbolic evidence extracted from sensor streams. This section makes that repositioning precise.

### 5.1. From Classifier to Interpreter

Comprehensive overviews of LLMs describe them, at their core, as models of the statistical structure of natural language, trained at a scale that induces broad general knowledge and general reasoning heuristics [54,55]. As Blank [54] notes, what LLMs are supposed to model—and what they in fact model—is a subject of active theoretical debate, but for our purposes, the operational point is that LLMs are exceptionally well-adapted to semantic operations over structured symbolic input: paraphrase, aggregation, contradiction detection, justification, and dialog.

This capability is exactly what the neuro-symbolic architecture of Section 4 needs downstream of its fuzzy affective/cognitive primitives. Rather than asking an LLM to classify a raw waveform or to ingest a pixel stream and produce a state label, we ask it to reason over a structured symbolic representation of what the sensors have already told us, “confusion regarding concept C is high, cognitive load is moderate, arousal has risen over the last two minutes, textual sentiment in the last dialog turn was mildly negative”, and to produce a coherent, contextualized interpretation with an explicit justification chain. Recent architectural proposals for perception–cognition–action pipelines in autonomous systems adopt precisely this pattern, using LLMs as the cognitive layer that mediates between perception and action rather than as the perception layer itself [56]. Analogous perspectives in medical research argue that LLMs are best used as reasoning aids over evidence extracted by domain-specific pipelines, rather than as end-to-end predictors [57]. To make this reasoning pathway concrete, Box 1 provides a hypothetical worked example showing how structured sensor-derived evidence can be transformed into an interpretable, evidence-grounded recommendation.

Box 1 **From sensor evidence to LLM reasoning—a worked example.**

Suppose an adaptive-learning system observes a learner solving a recursion exercise. At time *t* = 09:14:22, the sensor front-end and fuzzy primitive layer produce the following structured symbolic tuple:

  {

 learner_id: L_042,

 task_context: {module: “Recursion, item: 3.2, elapsed_s: 120},

 cognitive: {

  confusion(concept = “base_case”): 0.78 [rising, τ = 90 s],

  load: 0.65,

  engagement: 0.55 [falling, τ = 60 s]

 },

 affective: {

  arousal: 0.62 [rising, τ = 120 s],

  valence: −0.25

 },

 evidence: {

  EEG_theta_frontal: 12.4 μV (± 0.8),

  EDA_phasic_peaks_60s: 3,

  gaze_fixation_var: 0.42,

  text_sentiment_last_turn: −0.31,

  keystroke_pause_ratio: 0.71

 },

 learner_model_prior: {

  repair_hypothesis: “base_case_boundary_misconception (0.62)”

 }

}

  The LLM reasoner is prompted with this tuple and asked to (i) produce a concise interpretation, (ii) issue an instructional recommendation with a justification chain that references specific evidence fields, and (iii) report calibrated confidence. A characteristic well-formed output is:

  L_042 shows rising confusion on base_case, corroborated by rising physiological arousal and negative textual sentiment over the past 90–120 s (confidence: 0.74). Recommendation: present a worked example of a base-case boundary check, then re-check comprehension via a 2-item probe. Justification: the pattern is consistent with the repair-theory-consistent base_case_boundary_misconception prior (0.62) and with the falling-engagement trend.

  The output is (a) grounded in the supplied symbolic evidence, (b) accompanied by calibrated confidence, (c) contestable in dialog, and (d) directly actionable by the instructional layer. A rationale that references evidence that was not supplied—a common hallucination failure mode—is rejected by the symbolic-consistency check discussed in Section 5.5.

  *(Note on calibration: The asserted score* c=0.74 *is an illustrative hypothetical value instantiated from Section 5.3:* $c=\Phi \left(\sum_{i}w_{i}\mu_{i}q_{i}+\beta \ln\frac{P\left(H\right)}{1-P\left(H\right)}\right)$*. Parameterizing active primitives (* $\mu_{\mathrm{confusion}}=0.78,q_{1}=0.90,w_{1}=0.6$*;* $\mu_{\mathrm{load}}=0.65,q_{2}=0.85,w_{2}=0.4$*; prior* $P\left(H\right)=0.62,\beta =0.35$*) yields logit* z≈0.814*. Applying temperature scaling* $\Phi \left(z\right)={\left(1+e^{-z/T}\right)}^{-1}$ *with* T=0.78*—optimized on validation splits to minimize Expected Calibration Error (ECE)—yields* c≈0.74*.)*

### 5.2. Multimodal LLMs and Vision–Language Reasoning over Sensor Data

The rise in multimodal LLMs and vision–language models (VLMs) extends this repositioning naturally. Evaluations of VLMs on learning-engagement detection show that generic multimodal foundation models can already recover engagement signals from classroom footage that previously required bespoke engagement classifiers and—crucially—can accompany their inferences with natural-language justifications [31]. Recent perspectives on multimodal-sensing-enabled LLMs for automated emotional regulation identify the same architectural pattern as an emergent standard: sensor pipelines feed structured evidence into an LLM that then reasons about emotional dynamics and recommends regulation strategies [28]. Work on unlocking explainable and effective multimodal affective reasoning via LLMs formalizes the case: LLMs, when correctly conditioned on multimodal evidence, produce affective interpretations that are simultaneously more accurate and more explainable than opaque classifier baselines [53].

An important corollary is that the sensor-side design objectives shift accordingly. If the LLM will perform the interpretive work, the sensor front-end should be optimized to produce evidence that is legible to a reasoner—sparse, symbolic, well-calibrated, temporally annotated—rather than evidence that maximizes downstream classifier accuracy. This is a reversal of the assumption that has quietly dominated much end-to-end sensor-AI design.

### 5.3. Uncertainty-Calibrated LLM Reasoning

For human-state sensing, uncertainty is not a nuisance to be minimized but a first-class output. A system that reports “engagement dropped” without communicating how confident it is in that report or on which sensor evidence the report rests provides less actionable information than one that reports a graded, well-calibrated interpretation with its evidential basis exposed. Recent work characterizing LLMs as uncertainty-calibrated optimizers for experimental discovery demonstrates that LLMs can, under appropriate protocols, produce well-calibrated confidence estimates that are usable by downstream decision-makers [58]. Comprehensive reviews of neuro-symbolic AI similarly foreground uncertainty quantification as one of the core benefits of the paradigm [42].

To make this calibration operational, it is necessary to have a clear methodological distinction between possibilistic fuzzy membership degrees and probabilistic confidence. Fuzzy degrees of membership $\mu \in \left[0,1\right]$ a are ontological, assessing the graded intensity with which an individual manifests a given state ($\mu_{\mathrm{confusion}}\;=\;0.78$ denotes substantial, non-binary cognitive disequilibrium). Epistemic confidence $c\in \left[0,1\right]$ is strictly evidential, measuring the system’s calibrated certainty that its interpretive diagnosis is correct given current sensor reliability and contextual priors. In our pipeline, the reasoning engine does not generate confidence scores via ungrounded linguistic heuristics. Conversely, it combines three explicit parameters: fuzzy primitive activations *μ_i_*, modality specific signal quality indices $q_{i}\in \left[0,1\right]$, and symbolic learner priors *P*(*H*). Formally, the reasoning layer is a probabilistic aggregator with soft constraints:

$$c\;=\;\Phi \left(\sum_{i}w_{i}\left(\mu_{i}\cdot q_{i}\right)\;+\;\beta \log\frac{P\left(H\right)}{1\;-\;P\left(H\right)}\right)$$

where $w_{i}$ reflects empirical sensor reliability weights, *β* is the relative scaling weight of the historical prior odds, and Φ denotes a post hoc calibration mapping whose hyperparameters are empirically fitted to minimize expected calibration error (ECE) across validation partitions. Downstream calibration quality is systematically audited via expected calibration error (ECE) across partitioned confidence bins alongside Brier scores to penalize overconfident misattributions under ambient label noise.

Coupling these observations to the human-state setting yields a concrete design principle: the LLM-based reasoning layer should produce, for each asserted state, both a graded fuzzy assignment and a natural-language rationale, and both should be calibrated so that downstream users (clinicians, teachers, or the person themselves) can consume them without needing to infer the system’s true confidence from indirect cues.

### 5.4. Agentic and Dialogic Sensor Interpretation

A final repositioning follows. Once LLMs are used as semantic reasoners over sensor evidence, their operation becomes naturally dialogic. The system can, in principle, be interrogated by the user or by a domain expert about why it inferred a given state; it can revise its interpretation in light of user feedback; it can flag when the sensor evidence is insufficient to sustain an interpretation and request clarification. Our recent work on trust recalibration in AI dialogue and, specifically, on conversational repair strategies in ChatGPT-based interaction provides an empirical basis for this claim: dialogic interaction can actively shape and recalibrate users’ trust over time [59]. Related work on reflexive dialog-based explainability for human–AI collaboration formalizes the pattern as a design idiom and shows empirical benefits for user comprehension when explanations are adaptive and interactive [20]. Personalized recommendation and content generation architectures built around LLMs demonstrate that domain-conditioned LLM reasoning is deployable in real educational and interaction settings [60,61].

We take this to indicate that “agentic sensing”—sensor systems whose interpretive layer is mediated by an LLM-based reasoning agent who articulates justifications and engages in dialog—is a viable and structurally coherent extension of a neuro-symbolic paradigm, not an arbitrary add-on.

### 5.5. Deployment Constraints: Latency and Bounded Reasoning

Two constraints that we have thus far under-emphasized deserve explicit treatment because they are the constraints most likely to determine whether the architecture we advocate becomes deployable rather than merely defensible: real-time latency and hallucination.

Real-time latency is the primary physical bottleneck of the proposed pipeline. In particular, the whole end-to-end workflow shown in Figure 1b from raw sensor acquisition and feature extraction, through the fuzzy primitive layer and symbolic knowledge base, to the LLM reasoner suffers delays at each sequential stage of processing. In immersive HCI (Section 7.3), the tolerable latency for state-driven adaptation is on the order of 100–300 ms; in bedside clinical monitoring (Section 7.2), acute-response scenarios impose similar constraints. A frontier-scale cloud LLM in the reasoning position would violate these budgets by an order of magnitude. Three architectural choices mitigate this. First, small, task-tuned, edge-deployable LLMs can be used for the tight adaptation loops, reserving frontier-scale models for the slower explanation loop; recent perspectives on LLMs in perception–cognition–action pipelines for autonomous systems adopt precisely this decomposition [55], and comprehensive overviews of LLM design foreground scale-versus-latency trade-offs as a first-class engineering variable [54]. Second, symbolic updates can be made asynchronous with LLM invocation: the fuzzy primitive layer runs continuously and pushes updates to the reasoning layer only when a symbolic delta exceeds a threshold, decoupling the tight sensor loop from the LLM cadence. Scalable neural-symbolic stream fusion architectures give a template for this decoupling at the reasoning layer [47]. Third, the reasoning layer can abstain when the fuzzy input is insufficient to sustain a bounded interpretation, avoiding unnecessary generation; recent work on LLMs as uncertainty-calibrated optimizers demonstrates that principled abstention is feasible in practice [58].

The main cognitive risks of using foundation models for state reasoning are hallucination and semantic drift. LLM reasoners are not inherently immune to confabulation or unsupported extrapolations, even when they are operating over structured evidence rather than raw signals. Under adversarial or ambiguous inputs, the model can generate a plausible-sounding rationale that does not, in fact, correspond to the sensor evidence it was given. Three complementary safeguards constrain this failure mode within the architecture we advocate. First, the LLM’s output is required to reference specific fields of the symbolic tuple it was conditioned on (Box 1), and a symbolic-consistency check on the output rejects any recommendation whose justification cites evidence that was not supplied. This is a form of retrieval-augmented, evidence-grounded reasoning specialized to the sensor domain, closely related to the neuro-symbolic information-fusion approach recently developed for explainable RAG in mental-health applications [52]. Second, the reasoning layer’s outputs are constrained to a fixed vocabulary of psychologically motivated primitives (Section 4.2), narrowing the space of hallucinable content; comprehensive reviews of neuro-symbolic AI foreground precisely this intervenability property as one of the paradigm’s core benefits [42]. Third, the reflexive dialog layer (Section 6.3) serves as a live consistency check: when a user or domain expert contests a rationale, the discrepancy surfaces as a symbolic conflict that the reasoning layer must resolve, rather than as a silent inference error. Empirical work on trust recalibration and conversational repair strategies in AI dialog demonstrates that this mechanism materially reduces the practical impact of hallucination on user-facing accuracy [20,59], and tutorial-level guidance on the responsible use of LLMs in medical research develops the same design commitments for a clinical setting [56].

Taken together, these two constraints determine whether the architecture reaches deployment. A perspective that ignores them is a perspective that stops short of engineering.

## 6. Explainability as Design Constraint, Not Afterthought

The third pillar of the perspective we advance is that explainability, in intelligent sensing systems for human states, is not a compliance requirement to be added after model training. It is a design constraint that shapes the choice of sensors, the structure of representations, the architecture of the reasoning layer, and the interface through which the system communicates with users.

### 6.1. Why XAI Is Not Optional for Human-State Sensing

There is now a substantial body of work characterizing the theoretical and practical desiderata of explainable AI (XAI), including comprehensive treatments of what is known and what remains open [35,36,62], of interpretable representations as a theoretical foundation [63], and of the impact of specific explanation techniques on user comprehension and confidence [38,64]. Comparative analyses of popular local-explanation methods such as LIME and SHAP, particularly in clinically relevant domains, illustrate both the value and the fragility of post hoc explanations [65]. Our own work on gradient-based visual explainability methods across convolutional and transformer-based vision models and on the comparative evaluation of XAI techniques for deep learning in image recognition has documented that different explanation methods can support materially different narratives about the same model’s decisions [66,67]. Explainable AI has also been developed and evaluated for personalized image and video content analysis in social media contexts [23].

For sensor systems that infer states about people, however, the stakes are qualitatively different from those in generic classification. The output of the system is a claim about the person; downstream actions—clinical, pedagogical, workplace—are premised on that claim; and the person themselves is entitled to understand, contest, and refine it. Sensor-focused XAI work in health monitoring makes this argument forcefully, framing explainable sensor interpretation as a precondition for the deployment of AI in clinical monitoring rather than as a decorative feature [43]. Domain-specific studies in the emergency department confirm that clinicians act differently—and more safely—when interpretive models are accompanied by well-designed explanations [68]. Analogous work on interpretable models in industrial sensor contexts, such as the analysis of blackcurrant powders via machine learning [44], illustrates that the same principle carries across application domains. Multimodal fusion for Alzheimer’s disease detection is another example in which explainability of the fused sensor-and-imaging model is not optional but constitutive of clinical acceptance [69].

### 6.2. Interpretation Drift, Label Noise, and Epistemic Honesty

Two chronic features of human-state sensing make explainability harder—and more essential—than in generic classification. First, labels are inherently noisy: affective and cognitive ground truth is annotator-dependent, context-sensitive, and often self-reported. Recent work on interpretation drift under label noise shows that even well-designed explanation methods can produce systematically different explanations when the same model is trained under different noise regimes, without any change in accuracy [37]. Second, deployment conditions shift continuously, and explanations must be robust to these shifts; work on the consistent-interpretation properties of explainable models addresses this challenge at the model-training level [38].

The implication we draw is that explanations should be epistemically honest. An honest explanation reports both what the system inferred and what it did not have evidence for; it distinguishes between claims supported by strong multimodal agreement and claims supported by a single modality under favorable conditions. Our recent Agency-First Framework for human-centric interaction and evaluation of generative AI operationalizes this stance: the framework treats user agency, transparency, and evaluable rationales as first-class heuristics, not as post hoc additions [18]. The same intuition underlies neuro-symbolic proposals for explainable retrieval-augmented generation in mental health, in which the explanation is generated jointly with the interpretation, from the symbolic layer [52].

### 6.3. Reflexive, Dialog-Based Explanation for Sensing Systems

If the reasoning layer of an intelligent sensing system is an LLM-based agent (Section 5), then the natural form of explanation is not a static feature-attribution map but a reflexive dialog. Empirical work on reflexive dialog-based explainability for human–AI collaboration has shown that adaptive and interactive explanations improve user comprehension and calibrated trust relative to static one-shot rationales [20]. Related work on trust recalibration and conversational repair strategies in AI dialog provides the mechanism by which such dialogs stabilize a shared interpretation between the system and the user over time [59].

A rapidly growing literature is beginning to organize XAI within LLM systems specifically. Surveys of explainability for large language models chart the emerging landscape of techniques and evaluation criteria [70], and complementary reviews articulate the specific challenges of LLM-based explanation in high-stakes settings [71,72,73]. Practical treatments of XAI methods for LLMs—including domain-oriented explorations in transparency and trust [74] and integrated Python-based methodological toolkits [75]—indicate that the mechanics of LLM explanation are becoming more tractable. Three-level frameworks for LLM-enhanced XAI, mapping technical explanations to natural-language user-facing rationales, provide one concrete blueprint for the layered explanations that human-state sensing systems require [76]. Empirical work on the impact of explainability in LLM applications on user experience confirms that these design choices meaningfully affect real-world usability [77].

We read this literature as validating a stronger design commitment for intelligent sensing systems: the reasoning layer, the sensor-side evidence layer, and the explanation layer should be co-designed, so that every state inference the system asserts is accompanied by a natural-language rationale that (i) references the actual sensor evidence that produced it, (ii) reports its calibrated confidence, and (iii) is contestable by the user in dialog. Nothing less will support the deployment of these systems in the settings where they can do the best.

## 7. Perspective Vignettes

To illustrate the three-part architecture—neuro-symbolic foundation, LLM-based reasoning, co-designed explainability—we briefly sketch three deployment vignettes drawn from our own and adjacent research programs. Each vignette is intentionally short: our aim is to demonstrate concreteness, not to substitute for the domain-specific research each vignette would require.

### 7.1. Adaptive and Affect-Aware Learning Environments

Consider an adaptive learning environment for a programming course. A modern instance can plausibly instrument the learner with a webcam for facial affect and gaze, an ambient microphone for speech and paralinguistic cues, a wristband for EDA and PPG, and a keystroke/interaction logger for behavior. Recent work has demonstrated that multimodal analyses of learner cognitive and affective states during programming activities yield rich, action-relevant insights [26]. Multimodal perspectives on affective dynamics in intelligent tutoring systems similarly show that state trajectories, rather than instantaneous labels, are what pedagogy requires [25]. Deep fusion of visual and textual data for E-learning and multimodal emotion recognition using deep convolutional neural networks with adaptive content delivery illustrate the current state of the art on the classification side [5,78,79]. Complementary work integrating multimodal analyses of emotional and cognitive states to understand learner behavior underscores that the interpretive step is the differentiator [27].

Under the architecture we advocate, the front-end pipelines feed structured, fuzzy affective and cognitive primitives (confusion regarding concept C, cognitive load, engagement, frustration) into an LLM-based reasoning layer that (i) consults the learner model—including cognitive-diagnostic hypotheses drawn from repair theory [13] and cognitive-ability structure [14] and long-standing lines of automated cognitive-state reasoning [15]—and (ii) proposes an instructional intervention with an explicit rationale. Independent work on neuro-symbolic affective-aware personalization in virtual learning environments [50] and on multimodal-sensing-enabled LLMs for automated emotional regulation [28] converges on essentially the same three-part architecture; CEREBRAL provides a concrete instantiation on the multimodal emotion-recognition side [51], and self-supervised multimodal learning for cognitive-state inference from wearable streams supplies the sensor-side precursor [2]. Emerging systematic reviews of AI integration in higher education, including frameworks such as FACETS and SAMR, highlight the importance of transparency, ethical considerations, and pedagogically grounded implementation [80]. Foundational multimodal affect-detection work also remains directly relevant, since it clarified early that no single stream would carry the interpretive load [7]. Vision–language models are now being evaluated on the specific task of engagement detection in this setting [31], providing a natural entry point for the LLM reasoning layer described in Section 5.

Deployment-side evidence from our own group aligns with this direction. Fuzzy-weighted sentiment recognition provides a concrete on-ramp to the fuzzy primitive layer [12]; fuzzy memory networks and contextual schemas provide the mechanism by which the LLM’s responses are grounded in the learner’s evolving state [17]; and LLM-based intervention pipelines evaluated in the same educational setting indicate that the recommendation machinery is separately viable in practice [60,61]. Broader surveys of affective computing in intelligent tutoring reinforce the same architectural direction [10].

### 7.2. Human-Centric Clinical Monitoring

The clinical setting sharpens every requirement we have articulated. Continuous physiological monitoring is increasingly feasible at bedside and in ambulatory care; the interpretive gap is correspondingly acute. Systematic reviews of human-centric cognitive-state recognition using physiological signals across clinical application domains highlight the persistent trade-off between predictive performance and interpretability and identify explanation as a chief obstacle to deployment [3]. Explainable AI for sensor-signal interpretation in health monitoring has begun to organize the specific set of techniques that can bridge this gap [43]. Neuro-symbolic explainable retrieval-augmented generation is being explored in mental-health applications, motivated by exactly the reasoning-and-explanation coupling we advocate [52]. Multimodal fusion with explainable AI for Alzheimer’s disease detection illustrates the same architectural pattern in a neurodegenerative context [69].

In such a setting, the case for LLM-based reasoning is strengthened by the need to communicate interpretations to clinicians in natural language, to accept clinician feedback dialogically, and to reason across heterogeneous sources of evidence (bedside biosensors, imaging, records). Tutorial-level guidance on the use of LLMs for medical research now provides methodological anchors for such use [56]. Perspectives on explainability of LLMs specifically in healthcare stress the additional constraints that clinical deployment imposes [72]. Emergency-department pathway prediction work provides a concrete demonstration of clinically useful explainable AI derived from sensor and record data [68]. Comparative evaluations of gradient-based explainability across vision architectures inform the choice of explanation method for imaging modalities in this vignette [66], and general XAI comparative work such as LIME/SHAP in diabetes prediction highlights the need for explanation-method selection to be principled, not defaulted [65].

### 7.3. Immersive and Neuro-Adaptive Human–Computer Interaction

The third vignette is immersive HCI—augmented and virtual reality environments in which the system adapts to the user’s affective and cognitive state in real time. Wearable neuro-adaptive multimodal architectures such as our NAMI framework [21] and personalized multimodal signal processing for AR environments [22] illustrate the current state of the sensing pipeline. Multimodal recognition of user states for HCI adaptation provides a broader framing of the interpretive requirements [81]. Importantly, the operational utility of such systems goes beyond passive state monitoring to active, closed-loop feedback in brain–computer and human–machine interfaces (BCIs/HMIs) [82,83]. As described in the fundamental BCI frameworks [82] and HMI paradigms for motor rehabilitation [83], closing the sensory-adaptation loop necessitates that the inferred neural and cognitive fluctuations be fed directly back into system control mechanisms. Our architecture transforms continuous biosignals into calibrated states that are semantically grounded, ensuring that adaptive interventions such as dynamic scaling of task difficulty, real-time stimulus recalibration, or robotic motor assistance are appropriate, safe, and transparent to the user and practitioner. Emotion-recognition work in human–robot interaction, including our NAO-based studies, extends the same architecture from AR into embodied interaction settings [11].

In this vignette, the case for the neuro-symbolic + LLM interpretive layer is again architectural rather than incremental. The sensor stack is heterogeneous (physiological, ocular, inertial, ambient), the interpretation must be real-time, the state must drive concrete adaptations (task difficulty, guidance, virtual-agent behavior), and the user should be able to interrogate the system about why it adapted the way it did. Sensing micro-motion patterns via mmRadar and video for affective and psychological intelligence provides an emerging sensor primitive that fits naturally into the architecture [4]. Gait-based affect recognition, using on-body smart devices, is a further primitive that extends the architecture beyond seated interaction [32]. Multimodal biomarker frameworks organize the space of sensor primitives from which the fuzzy layer draws [30]. Recent surveys on the state of the art in multimodal emotion recognition [8] and in affective transformers based on biosignals [9] confirm that the sensor and encoder machinery have advanced substantially, but sensor advances alone are not enough to resolve contextual ambiguity, and the interpretive and reasoning layer appears to be a decisive discriminator for robust closed-loop interaction.

## 8. A Research Agenda: From Raw Signals to Understood States

We conclude the substantive portion of this paper with a compact research agenda. Each item is an operational restatement of a claim developed above; each is stated as a design commitment rather than a research question because the transition we advocate is at least as much an engineering one as a scientific one.

### 8.1. Grounded Representations as First-Class Outputs

Intelligent sensing systems for human states should produce, as their primary output, a grounded symbolic representation of the person’s state—not a class index. The representation should be structured (a set of fuzzy affective/cognitive variables), context-annotated (a task, a timescale, an environment), and revisable (an updatable user model). The neural pipelines feeding the representation should be evaluated by the quality of the representation they support, not by classification accuracy on isolated labels. Our own line of work on user modeling—cognitive-diagnostic modules based on repair theory [13], cognitive-ability leaderboards [14], automated symbolic reasoning of learner cognitive states [15], fuzzy cognitive-map policy models [16], and NAMI-style neuro-adaptive multimodal architectures [21]—offers concrete templates. Multimodal biomarker frameworks such as MEmoR provide an equivalent template on the affective side [30]. Recent proposals for neuro-symbolic multimodal emotion recognition with psychological constraints and metacognitive reasoning further specify what such a representation should look like [51].

### 8.2. Uncertainty-First Design

The default output shape of an intelligent sensing system should include a well-calibrated uncertainty estimate. Uncertainty should be produced at the symbolic layer (over the fuzzy variables that constitute the state), it should be propagated through the LLM-based reasoning layer, and it should be visible to end users in natural language. Comprehensive treatments of uncertainty quantification and intervenability in neuro-symbolic AI provide a research foundation [42]. Recent work on LLMs as uncertainty-calibrated optimizers demonstrates that the reasoning layer can, in fact, be made to produce usable calibration [58]. Multimodal-sensing-enabled LLMs for emotional regulation illustrate the setting in which uncertainty-aware reasoning is most needed [28]. Perspectives on LLMs in domains that couple perception, cognition, and action underscore the same requirement in autonomous systems [55].

### 8.3. Reflexive Explanation Protocols

Explanation should be treated as a protocol, not as a feature. The protocol should specify how the sensor evidence, the symbolic interpretation, and the natural-language rationale are jointly generated, how they are surfaced to the user, and how user feedback re-enters the reasoning loop. Reflexive, dialog-based explanation has been shown to improve user comprehension and calibrated trust [20]. LLM-specific XAI frameworks—from three-level explanation architectures [76] to LLM-explainability surveys [70], handbook treatments of LLM explainability in human-centered AI [71], and treatments of explainability in the age of LLMs for healthcare [72]—provide the technical substrate. Comprehensible-AI perspectives on multimodal state detection add the sensor-side counterpart [34]. Foundational XAI evaluation frameworks and comparative analyses of local-explanation methods further constrain the design space [35,36,62,65]. Our Agency-First Framework and complementary work on reflexive dialog-based explanations provide operational blueprints for the human-facing side of this protocol [18,20]. Explainability of LLMs and its impact on user experience should be treated as a design KPI, not as a research afterthought [73,74,75,77].

### 8.4. Benchmarks, Datasets, and Ethical Frames

Benchmarks for intelligent sensing systems should evolve to reward representation quality, explanation quality, and calibration—not only classification accuracy. Validation of the proposed architecture requires controlled ablation studies comparing the neuro-symbolic + LLM stack to standard end-to-end deep multimodal baselines under real-world domain shifts. Empirical evaluation should operationalize: (i) representation fidelity (e.g., alignment between fuzzy primitives and expert clinical/pedagogical diagnostic models), (ii) epistemic calibration, (iii) explanation utility, and (iv) interaction overhead. Crucially, our main thesis that intermediate symbolic grounding and semantic reasoning are required to bridge the signal-to-meaning gap is empirically falsifiable. It would be directly falsified by:

- Pure end-to-end multimodal foundation models consistently show equal or better out-of-distribution transfer, equal calibration and verifiable non-hallucinatory explanations that are equally auditable and actionable by domain experts without explicit symbolic or fuzzy constraints;
- The computational latency and token cost of intermediate fuzzy-to-symbolic mapping and bounded LLM reasoning negate any observed improvement in decision trust or adaptive intervention efficacy in real-time closed-loop deployments;
- Human users and domain specialists systematically ignore or do not benefit from dialog-based state explanations and contestation mechanisms relative to simple, unadorned probability scores.

Empirical benchmarking demands strict operational criteria, in addition to high-level validation protocols. In particular, future deployments should be assessed along seven orthogonal axes: epistemic alignment of reported confidence and empirical accuracy as measured by expected calibration error (ECE) and Brier scores; faithfulness of explanations as measured by whether LLM rationales accurately reflect the active fuzzy primitives vs. ungrounded confabulations; rate of symbolic contradictions as measured by how reliably cross-modal conflicts are surfaced; temporal stability penalty as measured by spurious high-frequency state fluctuations across contiguous windows; abstention accuracy as measured by the system’s ability to withhold attribution as signal quality degrades; user correction and repair rates as measured by the resolution efficacy of the reflexive dialog loop; and end-to-end execution latency as measured by edge vs. cloud reasoning tiers.

To demonstrate the practical implementation of this pipeline, let us look at a representative feasibility walkthrough over synchronized multimodal biosignal streams (e.g., continuous ECG, EDA, and behavioral logs). In the feature-extraction stage, the continuous ECG and EDA recordings are split into short sliding windows and filtered through signal quality checks to extract heart rate variability metrics and phasic skin conductance response peaks. These continuous indicators are compared with Gaussian membership functions in the fuzzy primitive stage to produce graded state primitives. Intermediate activations include increased arousal ($\mu_{\mathrm{arousal}}\;=\;0.72$) and emerging stress ($\mu_{\mathrm{stress}}\;=\;0.68$). This fuzzy tuple is combined with task metadata and user priors in the symbolic context stage, which defines a contextual baseline prior of $P\left(\mathrm{Stress}|\mathrm{Task}\_\mathrm{Context}\right)\;=\;0.60$. The structured evidence tuple is then fed to an edge-quantized reasoning model, which produces the auditable attribution of an acute stress response with an ECE-calibrated confidence of c = 0.76, justified by an explicit rationale linking autonomic sympathetic activation with depressed vagal tone. Finally, if the user disputes this reading by specifying that their state is intense focus rather than distress, the reflexive dialog loop ingests this feedback to revise baseline emotional sensitivity parameters in the persistent user model. This concrete mapping shows how each stage in the proposed architecture processes multimodal evidence without requiring ungrounded architectural abstractions.

Datasets should be documented with the label-noise regime under which they were annotated, since interpretation drift under label noise materially affects downstream explanations [37]. Because the systems we are describing observe humans continuously and infer states about them, ethical frames must be developed alongside the technical ones. Systematic mappings of AI integration in higher education highlight the acceptance and equity considerations that this raises for one class of deployments [80]; parallel considerations apply in clinical and workplace settings [43,68,72]. We take this to imply that the community should treat data protection, informed consent, and the person’s right to interrogate the system as first-class engineering requirements, on the same footing as accuracy and latency.

### 8.5. Sensor-Side Consequences

Finally, we note that the perspective we have developed has direct implications for sensor design itself. If the interpretive layer is expected to reason symbolically over structured evidence, then the sensor front-end should be optimized to produce evidence that is legible to a reasoner: temporally annotated, cross-modally synchronized, sparse where possible, and equipped with calibrated confidence and provenance. Design choices that maximize classifier accuracy at the cost of interpretive legibility should be re-evaluated. Emerging work on self-supervised multimodal learning for cognitive-state inference from wearable streams points in a compatible direction [2]. Foundational work on multimodal semi-automated affect detection provides the historical template [7]. Human-centered computational modeling of cognition [39] supplies the psychological constraints against which the sensor evidence should be evaluated.

## 9. Conclusions

We have argued that the next inflection point for intelligent sensing systems observing human affective and cognitive states lies not in richer sensors alone but in the interpretive layer that turns signals into states. The signal-to-meaning gap is real, structurally rooted in the mismatch between deep-only pipelines and the semantic, temporal, context-bound shape of human states and, increasingly, the binding constraint on deployment in healthcare, education, and immersive human–computer interaction.

Our personal thesis is that this gap is best closed by a co-designed three-part architecture. A neuro-symbolic foundation grounds continuous sensor evidence in psychologically meaningful fuzzy primitives, over which symbolic reasoning is well-defined. Large language models sit above this foundation as auditable semantic reasoners—not as opaque classifiers—producing structured, contextualized interpretations with explicit uncertainty and explicit rationales. Explainability is treated as a design constraint that shapes each of the previous two layers, operationalized as a reflexive dialog between the system and the human user.

The reorganization we advocate has consequences that reach back to sensor design itself: the sensor front-end should be optimized for the legibility of the evidence it produces to a downstream reasoner, not only for the accuracy of a proximate classifier. Its consequences also reach forward to evaluation: benchmarks should reward representation quality, calibration, and explanation, not merely classification metrics. And its consequences reach outward, to the humans the systems observe: the sensing systems we are building should be the kind that a clinician, a teacher, or the person themselves would recognize as helping them understand something, rather than as merely reporting a label.

We do not claim any of this is finished business. We argue that a key, complementary frontier in the field now lies in the reasoning-and-explanation layer, alongside advances in active perception. Taking this interpretive dimension seriously—architecturally, methodologically, and ethically—will be significant to advancing the next generation of intelligent sensing systems.

## Figures and Tables

**Figure 1:**
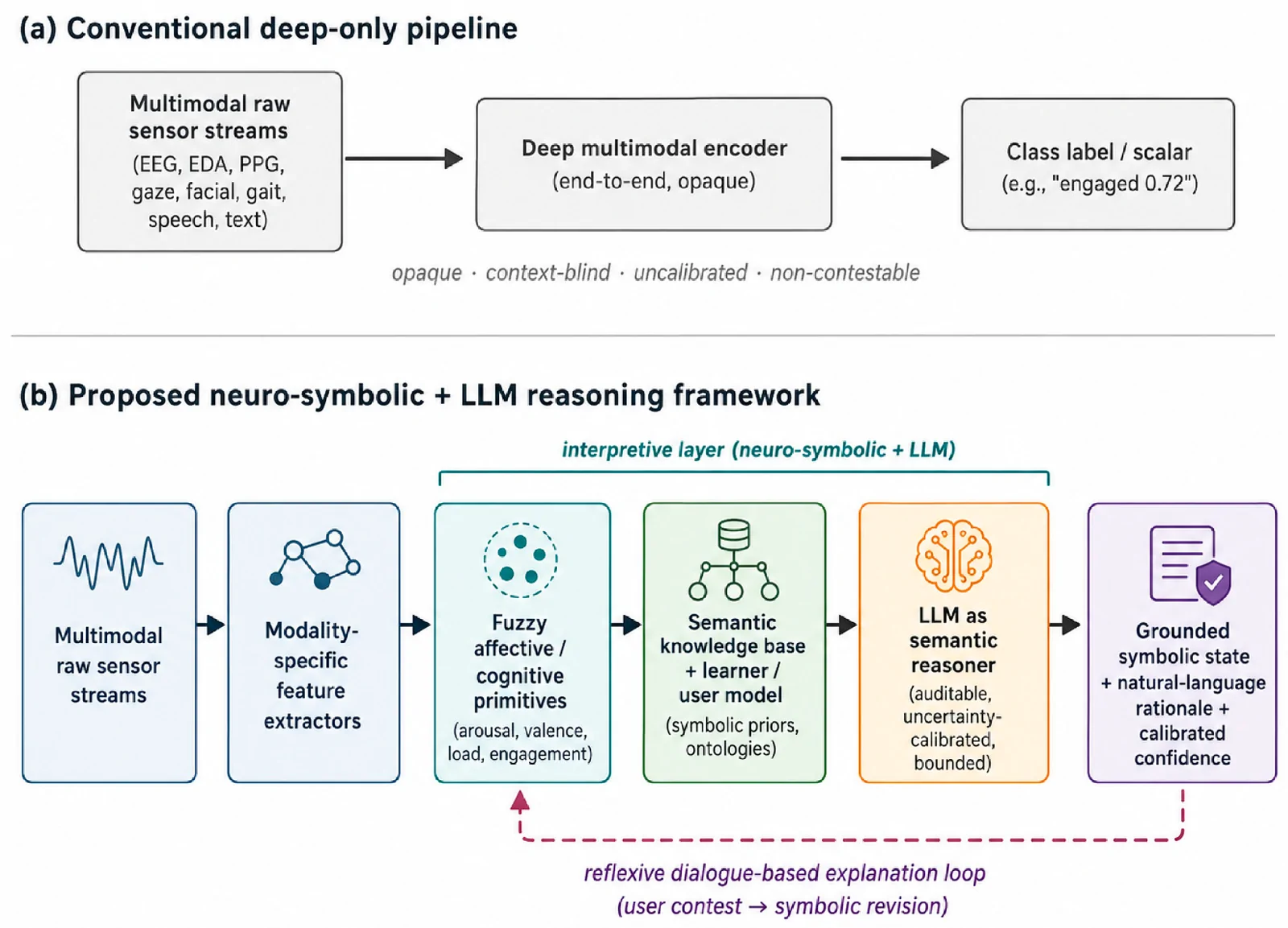
Comparison between (**a**) conventional deep-only end-to-end sensing and (**b**) the proposed neuro-symbolic + LLM interpretive framework. While constituent feature-extraction, fuzzy, and reasoning components draw on established empirical literature, their end-to-end integration into a reflexive, contestable loop represents a conceptual synthesis.

**Table 1:** Multimodal sensing landscape for human affective and cognitive-state estimation: raw signals, acquisition methods, spatiotemporal profiles, target constructs, and interpretive bottlenecks.

Modality Stream	Raw Signal and Physical Nature	Acquisition Sensors	Spatiotemporal Profile	Target Affective/Cognitive Constructs	Key Preprocessing and Interpretive Bottlenecks	Key Refs.
Central Biosignals (Neurodynamics)	Scalp microvolt fluctuations (μV), local field potentials	Multichannel EEG caps (wet/dry), fNIRS optodes	High temporal resolution (>250 Hz); coarse spatial localization	Executive load, mental fatigue, vigilance, cognitive workload, focused attention	Motion and ocular artifacts, non-stationarity, skull attenuation, volume conduction; poor individual-level interpretability	[3,9,24,29]
Autonomic/Peripheral Biosignals	Skin conductance (μS), pulsatile blood volume, bioelectric cardiac waveforms	EDA sensors (tonic/phasic), PPG optical sensors, ECG electrodes	Moderate-to-high temporal resolution (1–1000 Hz); spatially localized	Sympathetic arousal, autonomic tone, physiological stress, emotional valency correlates	Sensor displacement, sweat evaporation dynamics, baseline drift, motion artifacts; non-specific arousal attribution	[1,3,9]
Visual, Ocular and Kinematic	Pixel arrays (RGB/Depth), gaze coordinate vectors, 3D linear/angular acceleration (g, deg/s)	Video cameras, corneal-reflection eye-trackers, 6-DoF IMUs, mmWave radar	Moderate temporal (30–120 Hz); high spatial/positional granularity	Facial action units, fixation/saccade patterns, micro-gestures, postural slump, gait-based affect	Lighting sensitivity, occlusions, privacy intrusiveness, camera perspective drift; superficial behavioral mapping	[4,7,30,32]
Textual and Behavioral Interaction	Timestamps, keystroke/mouse trajectories, transcribed natural language tokens	Interaction loggers, keyboard hooks, speech-to-text microphones	Discrete, event-driven temporal dynamics; semantic spatiality	Conceptual confusion, frustration, semantic sentiment, task persistence	Sparse sampling during idle phases, conversational context dependence, sarcasm/ambiguity in language	[5,12,31,33]

**Table 2:** Comparative architectural positioning of related neuro-symbolic and LLM-based affective/cognitive sensing frameworks.

Framework/Study	Sensor Input Modalities	Intermediate Symbolic/Fuzzy Layer	Role of LLM/Foundation Model	Epistemic Uncertainty and Calibration	Explanation and Interaction Modality
Olaniyan and Wario (2026) [50]	Interaction logs, facial affect	Fuzzy cognitive rules and student ontologies	None (pure symbolic inference engine)	Crisp/fuzzy rule thresholds; no epistemic calibration	Static pedagogical rule firings
CEREBRAL (Kushwaha et al., 2026) [51]	Multimodal audio, video, text	Psychological knowledge graphs and commonsense priors	Metacognitive symbolic verifier (non-LLM)	Graph-based constraint satisfaction	One-shot static feature and rule attribution
Yu et al. (2025) [28]	Wearable biosignals, speech, video	Minimal/implicit intermediate semantics	Generative agent for emotional regulation strategy	Soft prompt-conditioned guidance (uncalibrated)	Natural-language conversational guidance (feed-forward)
Liao et al. (2026) [53]	Multimodal video, audio, dialog	Learned continuous multimodal embeddings	Direct multimodal affective reasoner and classifier	Softmax token probabilities	Natural-language post hoc rationale generation
Proposed Framework (This Work)	Central/peripheral biosignals, ocular, behavioral	Psychologically grounded fuzzy primitives and formal user models	Bounded semantic reasoner over structured symbolic tuples	Explicit possibilistic-to-probabilistic calibration (ECE-guided)	Reflexive, bidirectional dialog loop supporting user contestation and symbolic state repair

## Data Availability

No new data were created or analyzed in this study. Data sharing is not applicable to this article.
